# Continuing the success of *Journal of Endocrinology* and *Journal of Molecular Endocrinology*

**DOI:** 10.1530/JOE-23-0333

**Published:** 2023-11-29

**Authors:** Colin Farquharson, Ruth Andrew

**Affiliations:** 1 Roslin Institute, University of Edinburgh, Midlothian, Edinburgh, UK; 2 University/BHF Centre for Cardiovascular Science, Queen's Medical Research Institute, University of Edinburgh, UK

## Outgoing Editor-in-Chief

It is hard to believe it has been almost 6 years since I was invited to become co-Editor-in-Chief of both *Journal of Endocrinology* (JOE) and *Journal of Molecular Endocrinology* (JME). Due to their success, the number of submissions of both journals had risen sharply and in order to ensure timely decisions on all manuscripts, from submission to final outcomes, it was agreed that I would join as co-editor. This co-editor strategy worked and continues to work extremely well to the benefit of the journals, authors and readership and it has been my privilege to co-edit with both Sof Andrikopoulos and his replacement in 2021, Martin Haluzík. I thank them both for their professionalism and comradeship and their willingness to work together as part of a bigger team to safeguard the success of both journals. My tenure has been both enjoyable and professionally rewarding, during which time I have learned a great deal about the peer review process and scientific publishing. For this, I owe a great amount of gratitude to the publishers at Bioscientifica that I worked closely with and also the journals’ Senior Editors whose expertise have guaranteed that each accepted paper has gone through a rigorous peer-review process – a process that is essential to all scientific publishing to ensure the integrity and advancement of science.

There have been challenging times also. The coronavirus disease 2019 (COVID-19) pandemic brought many unforeseen adversities to laboratory research, scientists with school-aged children and early career investigators, amongst others. Paradoxically, like many other basic and translational journals, *JOE* and *JME* saw a growth in submissions in 2020, possibly reflecting the increased time to analyse and write original manuscripts and reviews during laboratory closures. There was also, of course, an increase in COVID-19-related submission and *JOE* and *JME* contributed to the dissemination of knowledge on COVID-19 and how it impacted endocrine function and homeostasis. Also, the experience shared by other publishers through the Committee on Publication Ethics (COPE) forum has made us aware of the dangers of ‘paper mill’ submissions to scientific enquiry and integrity. At *JOE* and *JME,* we have been taking steps to ensure submissions of this nature are deterred initially and identified at the earliest opportunity.

On a more positive note, in addition to publishing quality reviews, original research and anniversary issues, we have during my tenure also introduced some novel concepts and ideas. The Society for Endocrinology, with its flagship publication *JOE*, developed a leadership and development award programme that aims to identify and develop the future leaders of the Society. As part of this programme two awardees are given the opportunity to sit on the Senior Editorial Board as Early Career Editors. This gives them experience of peer reviewing (as editor and reviewer), hot topic selection, promotional activities and marketing feedback, and involvement with social media campaigns. We have also commissioned a ‘Rising Stars’ collection for *JOE* and *JME*. This is ongoing and our priority is to commission high-quality articles from early and mid-career researchers who are not yet fully established in their field. This we hope will provide additional recognition for them amongst their peers and help advance their careers.

Finally, it is time to sign off and hand over the baton to Ruth Andrew who I know will work with Martin Haluzík to ensure both journals go from strength to strength and maintain their position as leading global journals in endocrine research.

**Colin Farquharson**

## Incoming Editor-in-Chief

I am honoured to be chosen as the new co-Editor-in-Chief and very pleased to be supporting the Society for Endocrinology in this way. *JOE* and *JME* are flagship journals of the Society and Colin Farquharson and Martin Haluzík have nurtured them over a very challenging period. I am fully aware I need to fill ‘big shoes’ as Colin leaves and would be pleased to have Martin’s insight as I join him with the reins. I am full of enthusiasm while feeling the weight of responsibility on my shoulders, and I am hugely excited by the team with whom I will be working. The Editorial Board is replete with international leaders, with a passion for the journals, and have been hugely welcoming already.

Although we are now post-pandemic, we are still seeing the late effects with researchers re-establishing their fields of research, where for example animal colonies were stalled. Others have adapted by choosing new directions, for example exploring opportunities in bioinformatics and computational/data science. I think the time is right to widen the scope of the journals to embrace these avenues and reflect the value of interfacing wet and dry lab science in the preclinical field. There is also a timely opportunity to take a systems biology view of endocrinology as opposed to being organ driven and I hope we can embed editorial expertise to robustly assess such areas of systems biology as well as data science and systematic review.

To lead our field, reputation is of paramount importance, and robust, fair and timely review is an area close to my heart. Paper mills, as mentioned by Colin, create a major challenge in scientific publishing and we need to be on the front foot to establish ways to reassure readers of the integrity of data within our journals, for example working in line with research sponsor needs for data repositories. We need to keep abreast with what matters to researchers as they choose to publish. They have an increasing need for alternative modes of dissemination including to different audiences, for example the public, and these are areas we should be aiming to support to promote the valuable research in our journals.

The ‘open access’ landscape is still changing and it is important that our journals are welcoming and accessible to all. I have great faith in the Bioscientifica team in their knowledge to allow us to reach researchers worldwide, including initiatives to support researchers from low-/middle-income countries (LMICs) – such as Bioscientifica’s discount and waiver programme for authors in LMICs as defined by Research4Life (https://www.research4life.org/access/eligibility/). These are areas I will be monitoring keenly as the publishing landscape evolves.

Lastly, I hope I can bring perspectives from my previous roles as an editor and review editor for the *British Journal of Pharmacology* and specialty chief editor for *Frontiers of Endocrinology* – together these roles have given me a thorough appreciation of the challenges for ‘learned society’ journals and ‘born open access’ publishers. While differences exist in these models, I have found all the teams I have worked with have been united in ensuring the integrity and quality of publications of endocrine science and that is the mantra I shall carry forward into my new role in January 2024.

**Ruth Andrew**

